# New mutations found by Next-Generation Sequencing screening of Spanish patients with Nemaline Myopathy

**DOI:** 10.1371/journal.pone.0207296

**Published:** 2018-12-05

**Authors:** Sarah Moreau-Le Lan, Elena Aller, Ines Calabria, Lola Gonzalez-Tarancon, Cristina Cardona-Gay, Marina Martinez-Matilla, Maria J. Aparisi, Jorge Selles, Lydia Sagath, Inmaculada Pitarch, Nuria Muelas, Jose V. Cervera, Jose M. Millan, Laia Pedrola

**Affiliations:** 1 Genomic Unit, Health Research Institute Hospital La Fe (IIS La Fe), Valencia, Spain; 2 Genetics Unit, La Fe University Hospital, Valencia, Spain; 3 Research Group on Molecular, Cellular and Genomic Biomedicine, Instituto de Investigación Sanitaria La Fe, Valencia, Spain; 4 Biomedical Network Research Center for Rare Diseases (CIBERER), Madrid, Spain; 5 The Folkhälsan Institute of Genetics and the Department of Medical and Clinical Genetics, Medicum, University of Helsinki, Finland; 6 Unit of Rare Neuromuscular Diseases, La Fe University Hospital, Valencia, Spain; 7 Biomedical Network Research Center in Oncology (CIBERONC), Madrid, Spain; 8 Neuromuscular Diseases Unit, Neurology Department, La Fe University Hospital, Valencia, Spain, and Neuromuscular & Ataxias Research Group, Instituto de Investigación Sanitaria La Fe, Valencia, Spain; King Faisal Specialist Hospital and Research Center, SAUDI ARABIA

## Abstract

Nemaline Myopathy (NM) is a rare genetic disorder that encompasses a large spectrum of myopathies characterized by hypotonia and generalized muscle weakness. To date, mutations in thirteen different genes have been associated with NM. The most frequently responsible genes are *NEB* (50% of cases) and *ACTA1* (15–25% of cases). In this report all known NM related genes were screened by Next Generation Sequencing in five Spanish patients in order to genetically confirm the clinical and histological diagnosis of NM. Four mutations in *NEB* (c.17779_17780delTA, c.11086A>C, c.21076C>T and c.2310+5G>A) and one mutation in *ACTA1* (c.871A>T) were found in four patients. Three of the four mutations in *NEB* were *novel*. A cDNA sequencing assay of the *novel* variants c.17779_17780delTA, c.11086A>C and c.2310+5G>A revealed that the intronic variant c.2310+5G>A affected the splicing process. Mutations reported here could help clinicians and geneticists in NM diagnosis.

## Introduction

Nemaline Myopathy (NM) is a rare muscular disorder clinically and genetically heterogeneous which represents about 17% of all congenital myopathies [1]. The estimated incidence in the general population is one in 50,000 live births [2]. NM is characterized by hypotonia and generalized muscle weakness, more severe in the face, neck and limbs muscles. Although there is usually an overlap between different types, NM is clinically divided into six types depending on the onset and the severity of motor and respiratory involvement: severe congenital form which is the most life-threatening due to frequent early mortality; Amish form, typically fatal in early childhood; intermediate congenital, the most common type characterized by muscle weakness and feeding problems beginning in infancy; typical congenital, a less severe form of NM in which most of patients do not have severe breathing problems and are able to walk unassisted; childhood-onset form which usually causes muscle weakness in adolescent patients; adult-onset is the mildest form, usually related to muscle weakness development [3,4].

Diagnosis is based on clinical characteristics, the appearance of rod-shaped structures (also known as nemaline bodies) in muscle histology, and molecular genetic testing. To date, thirteen genes have been related to NM. Eight of these genes encode protein components of the muscle thin filament: *ACTA1* (1q42.13, MIM 102610), *NEB* (2q22, MIM 161650), *LMOD3* (3p14.1, MIM 616112), *TPM2* (9p13, MIM 190990), *TPM3* (1q21.2, MIM 191030), *TNNT1* (19q13.4, MIM 191041), *CFL2* (14q12, MIM 601443) and *MYPN* (10q21.3, MIM 608517) [5,6]. Other three genes are probably involved in the protein turnover in the muscle sarcomere via the ubiquitin proteasome pathway: *KBTBD13* (15q22.31, MIM 613727), *KLHL40* (3p22.1, MIM 615340), and *KLHL41* (2q31.1, MIM 607701). Two other genes have been related to NM: *TNNT3* (11p15.5) [7] and *MYO18B* (22q12.1, MIM 607295) [8]. Nevertheless, for some NM patients, mutations are not found in any of the known genes, suggesting that other causative genes are yet to be found.

NM can be inherited following an autosomal dominant pattern (familial or *de novo*) when *TPM2* and *KBTBD13* are involved, while most of other related genes have been described to follow a recessive inheritance pattern (*NEB*, *TNNT1*, *TNNT3*, *CFL2*, *MYO18B*, *MYPN*, *KLHL40*, *KLHL41*, and *LMOD3* genes). Heterozygous and homozygous mutations related to NM have been described in *ACTA*1 and *TPM3* suggesting these genes can follow an autosomal recessive or dominant inheritance pattern [9].

In this report we studied five Spanish patients with clinical and bioptical features suggestive of NM. *ACTA1* and *NEB* mutations were found. Clinical, histological, and molecular features are discussed.

## Methods

### Subjects

Five paediatric patients studied in the University Hospital La Fe (Valencia) and diagnosed with NM were recruited. They suffered from a congenital myopathy; the presence of rods in the biopsy was demonstrated in all patients using standard histological and histochemical techniques [10] (S1 Fig). The detailed clinical features in patients with NM were collected from medical charts. All subjects presented muscle weakness, hypotonia, delayed motor milestones and difficulties in swallowing, as described in Table 1. Genomic DNA and RNA from patients were extracted from peripheral blood samples following standard protocols. Samples from additional family members were used to perform segregation analysis of the sequence variants identified in the index patients. Written informed consent was obtained from all subjects and controls (or from their next of kin, caretaker or guardian in the case of minors/children). This study followed the tenets of Declaration of Helsinki and it was approved by the institutional board of the Ethics Committee of the University Hospital La Fe.

**Table 1:** Clinical features of patients diagnosed with NM included in this study.

Patient	1	2	3	4	5
**Congenital Form**	Intermediate	Severe	Severe	Typical	Typical
**Debut at birth**	+	+	+	-	-
**Sex**	Male	Male	Male	Male	Female
**Muscle weakness**	+	+	+	+	+
**Face**	+	+	+	+	+
**Neck flexors**	-	+	+	-	-
**Proximal limb muscles**	+	+	+	+	+
**Distal limb muscles**	-	+	+	-	-
**Hypotonia**	+	+	+	+	+
**Areflexia**	+	+	+	+	+
**Ophthalmoplegia**	-	-	-	-	-
**Bimodal communication**	+	+	+	-	-
**Normal Intelligence**	+	+	+	+	+
**Cardiac involvement**	-	-	-	-	-
**Bulbar weakness**	+	+	+	-	-
**Respiratory insufficiency**	-	+	+	-	-
**Walk independently**	-	-	-	+	+
**Wheelchair dependence**	+	+	+	-	-
**Cephalic support**	+	-	-	+	+
**Sedestation**	+	-	-	+	+
**Drooling**	+	-	-	-	-
**Scoliosis**	-	+	+	-	-
**Ogival palate**	+	+	+	+	+
**Ankle contractures**	-	-	-	-	+
**Articular hypermobility**	-	-	-	+	-
**PEG**	+	+	+	-	-
**Mechanic ventilation**	-	+	+	-	-

PEG: Percutaneous Endoscopic Gastrostomy Feeding; Bimodal communication (eyes, tongue and fingers). The presence of the clinical feature is symbolized by (+); no evidence of the symptom is represented by (-).

### Mutational analysis

Amplicon libraries were prepared using Ion AmpliSeq Neurological Research Panel (ThermoFisher Scientific) including 751 genes involved in neurological disorders according to the manufacturer’s instructions. This panel was used to screen the coding and intronic flanking regions of the following NM related genes: *ACTA1*, *CFL2*, *KBTBD13*, *NEB*, *TNNT1*, *TNNT3*, *TPM2* and *TPM3*. After preparation, the libraries were diluted to 100 pM and pooled in equal amounts. Sequencing was performed with an Ion Proton System using Ion PI HiQ Sequencing kit and the Ion PI Chip (ThermoFisher Scientific). Sequences were aligned against the human genome sequence (build GRCh37/hg19) with a Torrent Mapping Alignment program. After sequence mapping, the DNA variant regions were piled up with Torrent Variant Caller plug-in software and annotated with Ion Reporter v.5 software (ThermoFisher Scientific).

In the case of not finding any pathogenic variants, a second screening was performed using a commercial panel including two more NM related genes: *KLHL1* and *MYPN*. Capture libraries were prepared using ClearSeq Inherited Disease XT panel (Agilent Technologies) according to the manufacturer’s instructions. This panel includes 3,205 genes involved in rare inherited disorders. Sequencing was performed on a NextSeq500 (Illumina Technologies) with a Mid-Output flow cell for paired 150-cycle reads. In this case, sequences were aligned against the human genome sequence (build GRCh37/hg19) with BWA v.7.15. After sequence mapping, the DNA variant regions were piled up with GATK v.3.7–0 and annotated using ENSEMBL Variant Effect Predictor v.86 software.

Detected variants were prioritized according to:

- Functional impact on the protein (missense, nonsense, frameshift, in frame deletions or insertions, and splicing variants*). *Splicing variants will be considered up to ten nucleotides from the exon-intron boundary in intronic location, and up to four nucleotides from the exon-intron boundary in exonic location.
- Population frequencies (Minor Allele Frequency < 0.02) in databases as gnomAD (http://gnomad.broadinstitute.org/), ExAC (http://exac.broadinstitute.org/), 1000G (http://www.internationalgenome.org/) or in the *in house* IIS-La Fe Genomic Unit database.
- Presence in disease databases: ClinVar (https://www.ncbi.nlm.nih.gov/clinvar/), HGMD (https://portal.biobase-international.com), LOVD (http://www.lovd.nl/3.0/home).
- *In silico* predictions: SIFT (http://sift.bii.a-star.edu.sg/), PolyPhen-2 (http://genetics.bwh.harvard.edu/pph2/), Mutation Taster2 (http://www.mutationtaster.org/), NetGene2 (http://www.cbs.dtu.dk/services/NetGene2), NNSPLICE v0.9 (http://www.fruitfly.org/seq_tools/splice.html), Human Splicing Finder (HSF; http://www.umd.be/HSF3/index.html), and Spliceview (http://bioinfo.itb.cnr.it/oriel/splice-view.html).

Resulting variants were interpreted following the American College of Medical Genetics and Genomics (ACMG) guidelines [11].

All potential disease-causing variants detected in probands and their relatives were confirmed by Sanger sequencing using the Big Dye 3.1 terminator sequencing kit on an ABI 3500xl sequencer (ThermoFisher Scientific). In patient 4, regions with depth coverage under 20X were also studied by this method using specific primers for genomic amplification (S1 Table): exons 20, 21, 27, 57, 61, 74, and 149 of *NEB* transcript NM_004543; exons 84, 105, and 135 of *NEB* NM_001164508; and exon 1 of *KLHL41* (NM_006063).

In the case of not finding any pathogenic variants using Next Generation Sequencing (NGS) analysis, a conventional Sanger sequencing screening was performed in other three NM related genes not included in the NGS panels used previously: *LMOD3* and *KLHL40* (S1 Table).

Samples from patients 2 and 3 were analysed on a custom comparative genomic hybridization array targeting the known NM genes and 175 other genes related to neuromuscular disorders [12]. Moreover, a SNP array, CytoScan HD (Affymetrix), was used in patient 3. Labelling and hybridization were performed according to the manufacturer’s specifications (Affymetrix). Scanned array images were analysed by Chromosome Analysis Suite Affymetrix v.3.1 software (Affymetrix).

### Splicing analysis

Splicing analysis was performed to obtain insights into the pathogenicity of *novel* variants detected. RNA was extracted from peripheral blood samples following standard protocols and RT-PCR analysis was carried out by GeneAmp RNA PCR Core Kit (Thermofisher Scientific). cDNA was used as template in PCR reactions with specific primers in order to amplify and sequence regions of interest containing *novel* variants (Table 2). PCR products were tested on QiAxcel (Qiagen) and purified by Illustra ExoProStar 1-Step (Fisher Scientific). Experiments were performed in duplicate. For the protein nomenclature we used the Mutalyzer 2.0 beta-21 software (available at https://mutalyzer.nl/).

**Table 2:** Primers used to amplify fragments of cDNA.

**Gene**	**Exons**	**Primers**	**Sequence 5'-> 3'**	**Size** **(bp)**
***NEB*** **NM_001271208.1**	20–28	**NEB_20–28_EXT-D**	GCATTAATGACGATCCCAAGATG	929
		**NEB_20–28_EXT-R**	ACTTGGAGCATATCAAGAGGTGC	
		**NEB_20–28_INT-D**	GAACTTCAGTGAGGCTAGATATAAAGACT	550
		**NEB_20–28_INT-R**	CTTGTACATCACATCGCTGGTGTT	
	73–77	**NEB_73–77_EXT-D**	TACAGCAGCCCAGTGGACATGCTTG	682
		**NEB_73–77_EXT-R**	GGATCTTGGCCACATGGATGGACCAC	
		**NEB_73–77_INT-D**	TAGTGATACTATCTACCGTCAGCGT	261
		**NEB_73–77_INT-R**	GACTCTTCCAAAGCAAGTTTATAGA	
	111–114	**NEB_111–114_INT-D**	TCAACTTTACGCCTGTGGATGACAG	396
		**NEB_111–114_INT-R**	TCTCATTCTGAATCTGGCCTGCATGC	
		**NEB_111–114_EXT-D**	ACTGCAGAGCGATAATGTTTACAAG	751
		**NEB_111–114_EXT-R**	TCTGTAGACATTATCACTCTGTAGGTC	

EXT: primers used for amplification; INT: primers used for sequencing.

## Results

### Selected variants

NGS analysis detected five prioritized variants in *NEB* and *ACTA1* genes. Table 3 shows selected variants and their interpretation. Two previously reported mutations were detected in patient 2 and 5: c.21076C>T (p.Arg7026Ter) in the *NEB* gene (NM_001271208.1), and c.871A>T (p.Ile291Phe) in *ACTA1* gene (NM_001100.3). Moreover, three *novel* variants were detected in patient 1 and 4: c.17779_17780delTA (p.Tyr5927HisfsX17), c.11086A>C (p.Thr3696Pro), and c.2310+5G>A, in *NEB* gene. Patients 1 and 4 were both carriers of two pathogenic compound heterozygous variants but in patient 2 only one pathogenic heterozygous variant was detected. In this patient, a second non-pathogenic variant (c.23372T>C; p.Met7791Thr) was detected. This variant was previously described by Lehtokari *et al* in 2006 as pathogenic in compound heterozygous with another frameshift variant in one NM patient [13]. In 2016, Abouelhoda *et al* reclassified this variant as benign based on its frequency in the population of Saudi Arabia [14]. Despite its global low allele frequency in gnomAD (0.15%), this variant is not likely to be pathogenic because population data bases have shown that missense variants with low allele frequencies are common in *NEB* gene. Also, this variant is not located in the conserved actin- or tropomyosin-binding site. In this patient, we studied the presence of CNVs as a second disease-causing mutation but no CNV was detected in any of the targeted genes with these techniques.

**Table 3:** List of selected variants.

Patient	Gene	Sequence variants	State	References	ACMG evidence of pathogenity
**1**	*NEB*	c.17779_17780delTA	Het	*novel*	Path
		c.11086A>C	Het	*novel*	VUS
**2**	*NEB*	c.21076C>T	Het	Lehtokari *et al*, 2014[15]	Path
**4**	*NEB*	c.2310+5G>A	Het	*novel*	Path
		c. 17779_17780delTA	Het	*novel*	Path
**5**	*ACTA1*	c.871A>T	Het	Laing *et al*,2009[16]	Path

Het: Heterozygote; Path: Pathogenic; VUS: Variant of Uncertain Significance

In patient 3, no candidate pathogenic variants were found by either NGS, array and Sanger sequencing analysis.

S2 Table shows selected variants and their bioinformatic annotations relevant for their interpretation.

**Fig 1:**
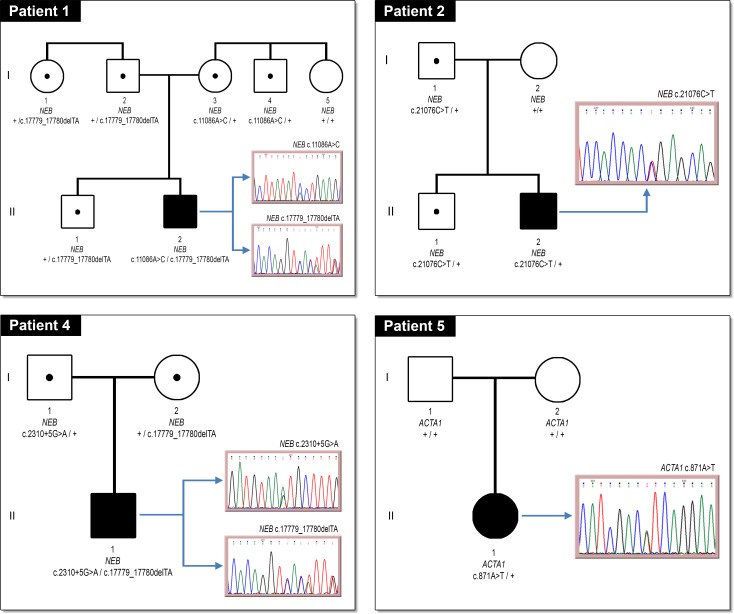
Segregation of selected variants. Pedigree symbols. Circle: female; square: male; filled: affected; unfilled: unaffected; spot: carrier; +: normal allele.

Pedigree and electropherograms for the four probands with *NEB* and *ACTA1* variants, and their family members, are shown in Fig 1. Patients 1 and 4 exhibited autosomal recessive inheritance pattern in the *NEB* gene showing two variants in compound heterozygosity. Patient 5 only showed one mutation in *ACTA1* gene suggesting an autosomal dominant inheritance pattern. Moreover, the mutation in this patient appeared to be *de novo*.

In order to establish the role of those variants in the pathogenity of the disease, *novel* variants were analysed with four *in silico* splice site predictors. Score–values were calculated ranking from 0 to 4 depending on the number of programs predicting splicing site alteration (Table 4). The intronic *NEB* variant c.2310+5G>A showed the highest score (4).

**Table 4:** Splicing prediction results in novel variants.

Patient	Sequence Variant (*NEB*, NM_001271208.1)	Type of Splice Site	*NetGene2*	*HSF*	*NNSplice*	*Splice View*	Score
**4**	**c.2310+5G>A**	**Donor**	**+**	**+**	**+**	**+**	**4**
**1, 4**	**c.17779_17780delTA (p.Tyr5927HisfsX17)**	**Acceptor**	**+**	**+**	**+**	**-**	**3**
**1**	**c.11086A>C (p.Thr3696Pro)**	**Acceptor**	**-**	**+**	**-**	**-**	**1**

Potential alteration of splicing is symbolized by (+). Neutral-effect predictions are symbolized by (-).

### cDNA sequencing confirms splicing alteration

**Fig 2:**
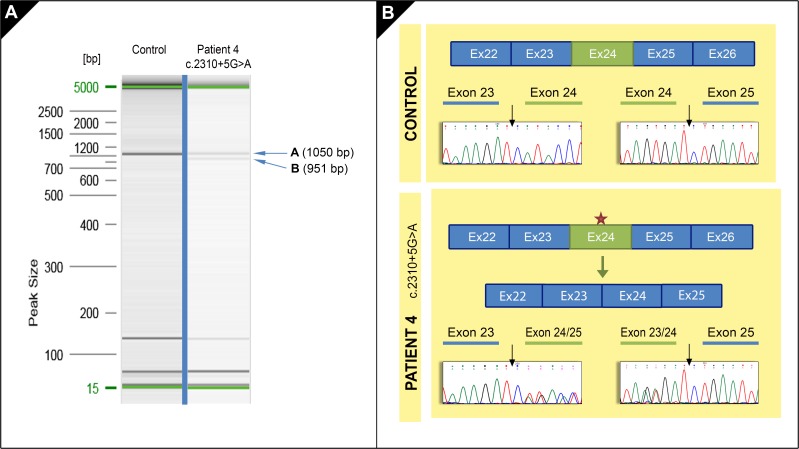
Splicing effects of *NEB* variant c.2310+5G>A. **A.** RT-PCR products obtained in patient 4 (band A and B) and in control sample (band A) are shown in this electrophoresis gel. **B.** Diagrams show the exons distribution in a cDNA fragment in a control sample and the effect of *NEB* variant (red star) in patient 4. Electropherograms show the *NEB* cDNA sequences in control and patient 4. In control, forward sequences show the junction in exons 23–24 and exons 24–25. In patient 4, forward sequence shows the junction in exon 23 and the overlapping exon 24/25; complementary reverse sequence shows the junction in the overlapping exons 23–24 and exon 25 suggesting the exon 24 skipping.

To confirm *in silico* predictions for the three *novel* variants (Table 4), a cDNA-based splicing assay was performed in patient 1 and 4 [17]. cDNA sequencing showed that only the intronic variant c.2310+5G>A (patient 4) was affecting the mRNA processing (Table 5). The other two variants did not show any alteration in this assay. RT-PCR product examination in patient 4 showed the presence of two different size bands: 1050 bp and 951 bp (Fig 2). The 1050 bp band had the expected size for the *NEB* transcript NM_001271208.1 (cDNA fragment corresponding to exons 20 to 28). The 951 bp band suggested a splicing alteration causing the appearance of a new isoform of *NEB* gene in which the exon 24 is skipped, as confirmed by Sanger sequencing. This exon skipping would lead to an in-frame deletion of 33 aminoacids from the protein creating a new protein: p.His738_Asp770del (c.2212_2310del).

**Table 5:** Effect of NEB *novel* variants on splicing.

**Candidate variants**	**Score (0–4)**	**Effect on mRNA processing**	**Effect on protein**
**c.2310+5G>A**	4	Exon skipping	p.His738_Asp770del
**c.17779_17780delTA**	3	No effect on splicing	p.Tyr5927HisfsX17
**c.11086A>C**	1	No effect on splicing	p.Thr3696Pro

## Discussion

In the present study, NGS sequencing in a small NM cohort was carried out. Two previously reported mutations and three additional *novel* mutations in *NEB* and *ACTA1* genes were successfully identified in four out of five patients.

The complexity of NM study is not only due to the clinical and genetic heterogeneity of the disorder but also to the peculiarity of the structure of *NEB*, the gene most frequently involved in the pathogenesis of the disease. This gene is one of the largest and most complex genes involved in neuromuscular disorders. Thus the traditional sequencing is time consuming and too expensive for this purpose. Moreover, the discovery of new related genes is increasing, making NGS approaches crucial for the optimal mutational screening of all known related genes in this disease. NGS sequencing was a rapid way to identify three *novel NEB* variants in two patients and other two previously reported mutations in *NEB* and *ACTA1* genes in two other patients (Table 3). In patients 1 and 4, the molecular cause of disease was found in the *NEB* gene while patient 5 showed a mutation in the *ACTA1* gene. These results are consistent with the literature as these two genes are the most frequently associated with NM disease [13,18]. Alterations found in this study include several types of missense mutations, small deletions, stop codons, and splicing variants.

*NEB* gene encodes nebulin, a giant protein of 600–900 kDa that comprises 183 exons. This protein is localized in the thin filament of the sarcomeres in skeletal muscle. Nebulin regulates actin filaments length, actin–myosin interactions and myofilament calcium sensitivity [19].

Nebulin contains actin-binding domains referred to as “nebulin repeats”. These repeats are ~35 amino acids in length and contain a conserved SDxxYK motif [20]. Within the central region of the molecule (repeats 9–162) groups of seven single repeats are arranged into “super repeats”, which also contain a single conserved motif (WLKGIGW; located at the end of the third repeat within each super repeat) and are thought to interact with troponin/tropomyosin complexes (calcium-mediated regulators of muscle contraction) throughout the actin filament [21,22,23]. *NEB* variants described in this study are located among the exons 24, 75, 113, and 140. We suggest that changes detected in *NEB* would probably affect repeats structure and therefore, the interaction with the rest of the skeletal muscle sarcomere proteins, altering its correct functioning.

A single functional allele of the nebulin has been described to be sufficient to preserve normal levels of protein, resulting in an unaltered skeletal muscle function [23]. The *NEB* gene, therefore, has a typical autosomal recessive pattern of inheritance, so patients necessarily must bear two mutations in compound heterozygosis or a homozygous mutation to develop the disease. In this work, we have classified those variants in compound heterozygosis in *NEB*, which meet the criteria mentioned in the material and methods section, to be disease-causing.

The *novel NEB* variant c.17779_17780delTA was found in two apparently unrelated families. This small deletion of two nucleotides probably produces a frameshift mutation leading to a premature stop codon in the protein. This variant was considered as pathogenic following ACMG guidelines [11].

The *novel NEB* variant c.11086A>C is a missense variant found in compound heterozygosity with the variant c.17779_17780delTA. This variant is located in a conserved actin-binding site and it is not found in population database gnomAD. This variant was considered as uncertain significance following ACMG guidelines [11].

The *ACTA1* gene encodes skeletal muscle α-actin which is the predominant actin isoform in the sarcomere thin filaments of adult skeletal muscle and it is essential, along with myosin, for muscle contraction. Mutations in *ACTA1* can interfere with the function, assembly and stability of the fine filaments resulting in different overlapping congenital myopathies, including NM [16]. A previously published mutation (c.871A>T; p.Ile291Phe) in *ACTA1* was detected in one patient. This heterozygous variant appeared *de novo* in this patient and was not found in gnomAD and ExAC databases supporting the pathogenic interpretation. According to our results, most described mutations in this gene are dominant and *de novo* [16].

Patients suspected of NM, underwent muscle biopsy and were diagnosed by the presence of nemaline bodies in the muscle fibers. In this study, genetic results have confirmed the histological and clinical diagnosis in a high ratio (three of five patients). As a first approach, a commercial NGS panel including the seven most frequently NM-related genes (*ACTA1*, *KBTBD13*, *NEB*, *TPM2*, *TNNT1*, *TPM3*, and *CFL2*) was used. In patients 1, 4, and 5, the molecular cause of the disease was found. However, no putative pathogenic variants were detected in patient 3. Therefore, a new NGS study was carried out in this patient including the *KLHL41* gene by using a new commercial panel available at this time in our laboratory. This panel also included the seven NM related genes previously studied. They were sequenced again with better depth coverage and they were reanalysed. After these NGS approaches, patient 3 did not show any potential pathogenic mutation in the eight studied genes. In order to achieve the complete sequence of all known NM-related genes, two more genes not included in the previous NGS panels, *LMOD3* and *KLHL40*, were Sanger sequenced by designing specific primers (S1 Table). Finally, to study the existence of large rearrangements in patient 3, a SNP array and a custom comparative genomic hybridization array [12] were performed without additional findings. Unfortunately, patient 3 remained undiagnosed at a molecular level. Patient 2 is carrier of one heterozygous pathogenic nonsense mutation in *NEB*, and the second disease-causing mutation remains to be identified. We suggest the second mutation might be a large rearrangement or a deep intronic mutation not detected by these techniques. Sequencing technical limitations comprises the detection of deep intronic variants or variants located in regulatory regions since these regions are not included in the NGS panels and nor in Sanger sequencing design. In addition, changes in CG-rich regions are also difficult to detect and mutations in these regions could have gone unnoticed [24]. Moreover, for some NM patients, mutations are not found in any of the known genes suggesting that other causative genes remain to be found and a deep variant reanalysis should be done as soon as new genes are described to be related to the disease.

Recently, *MYO18B* have been associated to NM with cardiomyopathy [8] even if NM is not typically associated with these disorders. This gene has not been sequenced in the present work because none of our patients presented cardiomyopathy and thus we did not expect to find the molecular cause of NM in this gene.

Pathogenity of the three *novel* variants was *in silico* predicted (Table 4) and score values were assigned to them. The cDNA-based splicing assay revealed that only the variant with the highest score, c.2310+5G>A, affected the splicing process (Table 5).

However, the other two *novel NEB* variants, c.17779_17780delTA and c.11086A>C, obtaining scores of 3 and 1 respectively, did not show any effect on the splicing process when they were analysed by cDNA sequencing. According to these results, we suggest bioinformatic tools are not enough to assess the pathogenicity of *novel* variants and cDNA sequencing must be performed to confirm their splicing effect on mRNA processing.

In conclusion, NGS sequencing of known NM genes is useful for finding pathological variants in a selected group of NM patients. We showed that, in case *novel* variants were detected, especially those located near the exon-intron boundary, cDNA sequencing could be successfully performed to detect their putative effect on splicing.

## Supporting information

S1 Fig Muscle biopsies analysis from patient 1 by conventional microscopy. A. H&E: There is an increase in fat and connective tissue, internal nuclei and a wide variation in fibre size. B. Shows the presence of abundant rods, as red-staining structures with the Gomori trichrome stain, located mostly in the cytoplasm and often forming clusters.
(TIF)

S1 Table Primers used to amplify the specific regions. (PDF)

S2 Table List of selected variants and their bioinformatic annotations relevant for their interpretation. (PDF)
